# Database of pharmacokinetic time-series data and parameters for 144 environmental chemicals

**DOI:** 10.1038/s41597-020-0455-1

**Published:** 2020-04-20

**Authors:** Risa R. Sayre, John F. Wambaugh, Christopher M. Grulke

**Affiliations:** 1 U.S. Environmental Protection Agency, Center for Computational Toxicology and Exposure, 109 T.W. Alexander Drive, Research Triangle Park, NC 27709 USA; 2 Oak Ridge Institute for Science and Education, Oak Ridge, TN USA; 3 Department of Environmental Sciences and Engineering, University of North Carolina at Chapel Hill, Chapel Hill, NC USA

**Keywords:** Databases, Time series, Toxicology, Pharmacokinetics, Environmental sciences

## Abstract

Time courses of compound concentrations in plasma are used in chemical safety analysis to evaluate the relationship between external administered doses and internal tissue exposures. This type of experimental data is rarely available for the thousands of non-pharmaceutical chemicals to which people may potentially be unknowingly exposed but is necessary to properly assess the risk of such exposures. *In vitro* assays and *in silico* models are often used to craft an understanding of a chemical’s pharmacokinetics; however, the certainty of the quantitative application of these estimates for chemical safety evaluations cannot be determined without *in vivo* data for external validation. To address this need, we present a public database of chemical time-series concentration data from 567 studies in humans or test animals for 144 environmentally-relevant chemicals and their metabolites (187 analytes total). All major administration routes are incorporated, with concentrations measured in blood/plasma, tissues, and excreta. We also include calculated pharmacokinetic parameters for some studies, and a bibliography of additional source documents to support future extraction of time-series. In addition to pharmacokinetic model calibration and validation, these data may be used for analyses of differential chemical distribution across chemicals, species, doses, or routes, and for meta-analyses on pharmacokinetic studies.

## Abstract (summary)

**Table:** 

Measurement(s)	pharmacokinetic parameter • area under the plasma concentration time curve • Pharmacokinetic Parameter Numeric Result in Standard Unit • time sampled measurement data set
Technology Type(s)	digital curation • Algorithm
Factor Type(s)	species from which data were extracted • administration route • dose
Sample Characteristic - Organism	Rattus norvegicus • Homo sapiens • Mus musculus

Machine-accessible metadata file describing the reported data: 10.6084/m9.figshare.12000141

## Background & Summary

When assessing chemical risk, the U.S. National Research Council has delineated two aspects that must be considered: toxicological hazard and exposure^1^. Toxicological hazard may be conceptualized as the dose needed to cause an adverse effect, while exposure can involve the chance of occurrence, duration, route, and aggregate dose received. As hazard is being estimated more frequently using New Approach Methodologies (NAMs) to determine concentrations at which bioactivity occurs, a third component, toxicokinetics (TK), is needed to compare these hazard surrogates to exposure^2^. TK describes the absorption, distribution, metabolism, and excretion of a chemical within the body for a given species. Knowledge of TK allows translation of toxicological information, which might be collected in model animal species or *in vitro*, to humans or sentinel ecological species^3^. TK is also needed for translating external exposure doses into target tissue concentrations and vice versa (i.e., reverse dosimetry), allowing a linkage between cellular pathway perturbations and exposure amounts^4,5^. Detailed information on test animal species can be helpful, since there is uncertainty determining the relevance of an internal dose found in an animal TK study to humans, due to the higher doses generally used in this type of testing and other factors^6^. Although it can be useful in some contexts to differentiate between toxicokinetics and pharmacokinetics (PK), the two terms will be used interchangeably in this document.

Predicting internal doses is a primary task of 21^st^ century toxicology^7^. While pharmaceuticals are vetted with human clinical trials, and food additives or pesticides are tested using animal models, humans are also potentially exposed to many of the thousands of commercially-available chemicals (and their transformation products) for which there are limited toxicological and TK data^8,9^. To address this gap, computational modelling of TK can be performed based upon inputs from *in vivo, in vitro*, and *in silico* studies. *In vitro* tools have been developed to allow screening of chemical-specific TK properties for libraries of chemicals^9,10^. However, each input parameter carries an amount of uncertainty, which may not be readily quantifiable (for example, human genetic variation^11^). This uncertainty, combined with different underlying assumptions that may have formed the basis of the model, makes interpretation of the relevance of a model’s results to human health risk assessment complex^12^.

There is increasing acceptance of the use of *in vitro*-derived TK for chemical risk prioritization^13,14^ and the design of human clinical trials^15^. However, there is still need for careful model evaluation to determine which chemicals these techniques might work for, and how well^16,17^. Investigating every premise of a model would be time-intensive, and still would not demonstrate whether a modelling approach provides a unique description of the system modelled or an accurate prediction of dose-response curves in any non-modelled scenario^18^. While systems do exist for understanding the confidence with which *in vitro*-*in vivo* extrapolation (IVIVE) may be applied to pharmaceuticals (*e.g*., Riede, *et al*.^19^), it is simpler and more informative to compare modelling results with at least one observed instance of a relevant *in vivo* exposure scenario^14,15,17^. Unfortunately, the relative lack of structured, non-pharmaceutical *in vivo* pharmacokinetic data makes systematic evaluation of the performance of IVIVE for environmental chemicals difficult^17^.

An international workshop held in February 2016 focused on key steps needed to facilitate the adoption of high throughput TK into chemical risk prioritization and decision making^13^. That workshop recommended the “Creation of a database that could house all shared *in vitro* and *in vivo* TK data, and identification of actions to be taken to encourage sharing of existing data”^13^. Preliminary efforts by Wambaugh, *et al*.^14^ attempted to combine concentration vs. time (CvT) data collected from literature studies with data derived from new pharmacokinetic experiments creating a dataset covering 45 analytes. Here, we present a public database for storing CvT data and its associated study metadata for 187 analytes across more than 550 studies. In addition, the compiled CvT data have been analyzed with TK curve-fitting software to add a set of uniformly estimated properties, such as volume of distribution and elimination half-life, to the database. Database creation was initiated as a proof-of-concept designed to consider optimal structure, constraints, and detail required to facilitate “International harmonization of data requirements by regulators”^13^. Moreover, it provides an excellent source to evaluate modelled relationships between external and internal doses. It is hoped that these data will serve as a catalyst for the public sharing of curated TK data to improve assessment of risk posed by chemicals to human health.

## Methods

**Fig. 1:**
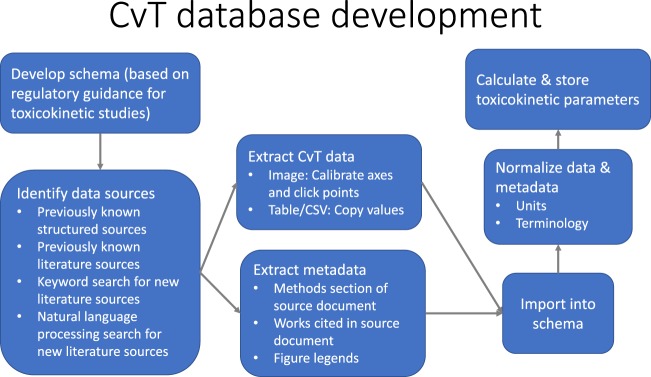
Workflow of CvT database extraction, collection, and calculation efforts.

The CvT database is intended to serve as a comprehensive repository for TK/PK data, including published sources of CvT data, CvT results, and derived pharmacokinetic parameters. While many TK experiments have been published, there has been little effort to normalize, structure, and centralize the results of these studies. The workflow in Fig. 1 outlines the steps performed to generate a compendium of CvT data including the collection of source materials, extraction of measured concentration time-series, and toxicokinetic parameters estimation.

### CvT data sources

As had been previously reported^13^, we were unable to find many structured sources of CvT data in the public domain. The two structured sources we found provided an excellent starting point to quickly gather content into our database; however, the majority of available TK CvT data is embedded in documents, typically journal articles. To develop a dataset that was sufficient to evaluate TK modeling methods for a broad range of chemicals, we realized that identification and extraction of primary TK literature data would be required, which we executed with a combination of custom machine learning and manual curation techniques. However, we recognized that the breadth of data contained in literature would likely be too large to address systematically with our limited curation resources. As a pilot of our data collection methods, we first focused on discovering PK data for a set of 351 environmentally-relevant candidate chemicals derived from the 2012 TSCA Work Plan^20^. Inclusion in this list suggests these chemicals had been identified as having enough available data to support development of evidence-based risk assessments, so we considered it likely that PK information would be available.

### Structured CvT data sources

**Wambaugh**, ***et al***.^14^ assembled a set of pharmacokinetic time-series data from literature source extraction and newly generated experimental data. This data for 45 analytes was available as a supplementary file (spreadsheet) to the original publication.

**Chemical Effects in Biological Systems (CEBS)**^21^, a public database hosted by the National Toxicology Program (NTP) contains a total set of 83 NTP-conducted TK studies covering over 40 test substances in rat or mouse gavage or IV studies. Although these are not peer-reviewed findings, they have undergone extensive quality assessment. The resultant data were exported from CEBS to Microsoft Excel files from the FTP site (ftp://anonftp.niehs.nih.gov/ntp-cebs/datatype/NTP_TK/Individual_Animal_Data/).

### Literature sources

In preliminary queries of PubMed, we quickly were able to identify sources of CvT data; however, finding extractable CvT content for our initial set of chemicals derived from the 2012 TSCA Work Plan proved more difficult than expected.

Querying PubMed using Entrez and MeSH terms is a common way to identify articles of interest. Therefore, using the Python 3 package BioPython^22^, Entrez searches were programmatically completed to identify all articles containing the name or CAS-RN for a compound of interest and any MeSH term containing “Dose-Response Relationship”, which was the most common term in the articles we had collected in a previous effort^23^. This yielded a list of 20970 PubMed articles. Of these, 549 publications that included names of higher-priority chemicals from the TSCA Work Plan were manually reviewed, but only 4% (22 articles) contained usable concentration vs time results (true positives, or TP), which made it clear that filtering by keyword search was insufficiently precise to efficiently identify relevant papers. This led us to investigate the use of natural language classification modeling to improve our workflow.

During the review of the Entrez search articles, we manually classed publications as either positive (22) or negative (527) for containing extractable time-series data. To this set we added 62 positives that we identified in earlier work^23^ yielding 84 total positives. This set was used to develop a predictive model for article relevance using Natural Language Classification (NLC). The abstracts of the publications were first filtered to remove extremely common words (Stanford stop words) and words that occurred with low frequency (defined as having a count value in the lowest quartile) to reduce data noise and computational time^24^. The cleaned abstracts were represented by TF-IDF (term frequency–inverse document frequency) matrices calculated using the Python 3 package ScikitLearn^25^. A suite of classifiers (Naive Bayes, Multinomial Naive Bayes, Bernoulli Naive Bayes, Logistic Regression, Stochastic Gradient Descent, Support Vector, Linear Support Vector, Nu-support Vector, K-nearest neighbors (3 and 5), Decision Tree, Random Forest, Multi-layer Perceptron, and AdaBoost) from ScikitLearn were trained to classify the sets with 10-fold cross validation. The negative set was undersampled to maintain balance. Results from all models that categorized at least 90% of abstracts correctly were averaged to create a consensus classification for all PubMed abstracts containing compound names of interest (CAS-RN was not used as an identifier in this trial, since it did not significantly improve recall). The approach yielded a much higher true positive rate (details in ***Technical Validation***). Natural language classification code is available in the information file on GitHub (CvT_find_papers.py).

### CvT time series

#### Structured source data extraction

**Fig. 2:**
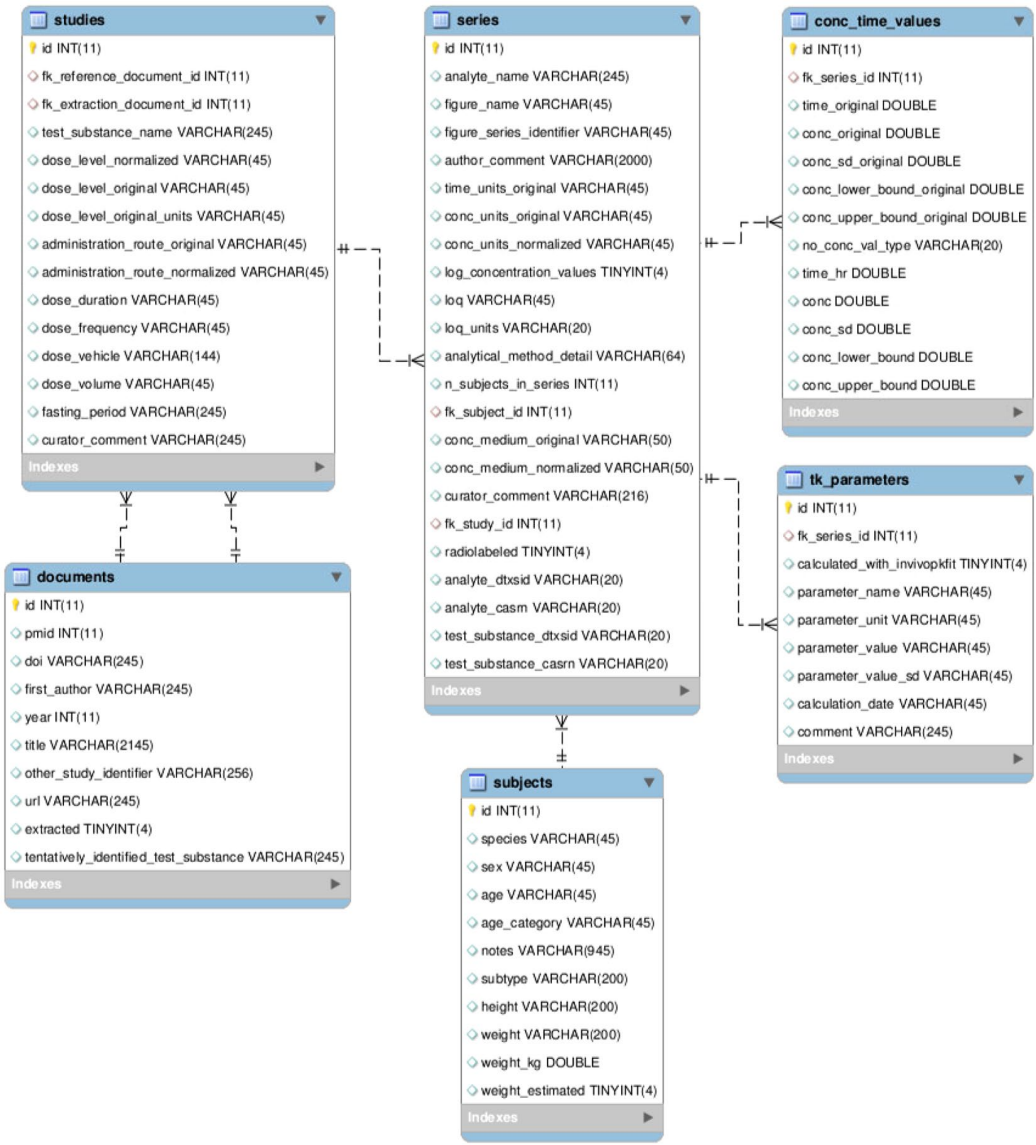
Entity-relationship diagram of CvTdb. The schema can be created with the file build_cvt_db.sql included in the figshare and GitHub.

Structured data was formatted into our data storage model shown in Fig. 2. For the Wambaugh *et al*. set, additional metadata was curated from the source documents cited in their publication. The CEBS set was parsed and loaded into the database using a combination of custom Python scripts and manual interventions due to the complexity of the provided data. Metadata such as fasting state were not readily available for CEBS records.

#### Literature source data extraction

Access to articles containing time-series data was provided by the EPA library, or by publishers posting works openly online in Adobe PDF format. Few publications made tabular concentration vs time data available. For most, the data was only available in plots. These plots were converted to images using the native screenshot tool in Windows 10. Collection of data was completed through WebPlotDigitizer^26^, a computer assisted program, requiring the identification of axes and scales for calibration and the selection of each point for extraction by a user. Automated approaches for this task are available, but were found to be error prone in calibration, identification of units and scale, or incapable of extracting data from plots with logarithmic axes. Extracted tables of concentration vs time data were exported from WebPlotDigitizer to CSV, then imported into the database using MySQL Workbench. Collecting the details of the study from which the concentration vs time data resulted was completed manually. Annotations as described in **Data Records** were manually curated from the Methods section of each paper. When available, TK parameters were also extracted from a publication’s Results section.

#### Time-series data normalization

The data were extracted with an effort to be as faithful to the original as possible. That means that everything was collected as reported, with no consideration for standardizing the units nor controlling the vocabulary used to describe the experiment. Standardized values were calculated and stored alongside the original values to facilitate easier comparison across experiments. These normalized concentrations are stored in µg/mL for tissues, excreta, or plasma (µgEq/mL for radiographic measurements), and µg/m^^3^ for breath. Times are stored in hours. A maximum of 5 numbers after the decimal point were stored for these data types. Doses are stored in mg/kg. In cases where the mass of the subject was not reported, and the dose was administered as a simple mass and not a body weight proportion, a mg/kg dose was calculated using the average mass for all subjects of that type. The names for the tissues in which doses were measured were standardized to the preferred names in NCI thesaurus as of 12 December 2018^27^. All subject measurements were stored in cm for height and kg for weight/mass. The subject age may be reported as a numerical value, a category, or both. When only a numeric value was provided, a category was inferred as given in Table 1.

**Table 1:** Age categories used for potential comparison across species.

	infant	child	adolescent	young_adult	adult	aged	unit
dog	0.75	1.5	6	12	24	100	month
human	0.08	2	12	16	21	60	year
mouse	1	3	5	7	10	45	week
nonhuman primate	0.5	6	36	48	72	240	month
rat	1.25	4	7	12	25	200	week

Values represent the lower threshold for age inclusion in this category. Younger than infant was categorized “neonate”. Categories were based on weaning age, onset and cessation of reproductive potential, and cessation of skeletal growth.

### Toxicokinetic parameters

Toxicokinetic parameters are often used as surrogates for more complicated time-series data when prioritizing chemicals according to their TK profiles; however, the models used to derive these parameters can be inconsistent between different publications, making direct comparisons problematic. Hence, a systematic extraction of reported TK parameters from literature was not done as part of this databasing effort (though several TK parameter estimations reported in the literature were gathered). Rather, the R package invivopkfit v1.5 was used to calculate pharmacokinetic parameters using the function fit_all as described in previous publications^14,28^. This package was used to fit the mean of all series from each plasma study to a one- or two-compartment PBPK model. Compartmental model analysis allowed all data from both intravenous (IV) and oral routes to be jointly analysed, when data from both routes was available. For the one-compartment model, the volume of distribution (V_d_, L/kilogram bodyweight, or L/kg BW) and the first-order elimination rate constant (k_elim_, 1/h) were estimated. For the two-compartment model, the primary compartment volume (V_1_, L/kg BW), inter-compartment exchange rate constant (k_12_, 1/h), and k_elim_ were estimated. For chemicals dosed orally, a gut absorption rate (k_absorb_, 1/h) and fraction of the oral dose bioavailable (F_bio,_ %) are also calculated. If no oral dosing data were available, only quantities that can be estimated from IV dosing were estimated (i.e., k_absorb_ and F_bio_ were not estimated). An aggregate set of these parameters were computed for all plasma concentration data for each administration route:test substance pair, as described in Wambaugh, *et al*.^14^.

The optimized likelihood for both the one- and two-compartment models were compared using the Akaike Information Criterion (AIC)^29^. Study-specific standard deviations were included in the number of parameters used to calculate AIC (e.g., if there were data from two studies, there were two standard deviation parameters estimated and factored into the AIC). The results of the model with the lesser AIC^30^ are stored in the database. The script used to calculate the parameters is publicly available through GitHub repository USEPA/CompTox-ExpoCast-invivoPKfit.

## Data Records

The collected data have been made publicly available on figshare as a zipfile containing a sql dump of the database^31^. These data, along with additional supplements, are also available at the GitHub repository https://github.com/USEPA/CompTox-PK-CvTdb, along with a xlsx template for submission of new studies to the database and the latest versions of the supplements to this paper. In addition, display of the data through the U.S. EPA’s Chemicals Dashboard (https://comptox.epa.gov/dashboard)^32^ is planned, but not yet implemented. The data is maintained by U.S. EPA’s Center for Computational Toxicology and Exposure in the data model depicted in Fig. 2 instantiated in a MySQL 5.6 community edition relational database of simple data types (text, numeric, and Boolean).

Provided are three subsets of data for use by the community:

1. Bibliography of journal articles suspected to have extractable CvT data (***CvT data sources***, provided within the SQL database and as a CSV file)
2. Extracted CvT data (***CvT time-series***, provided within the SQL database)
3. ***Toxicokinetic parameters*** (provided within the SQL database)

### CvT data sources

Identifying a set of sources for CvT data extraction was a key preliminary step in the development of this database. Once methods were developed to identify sources for our chemical domain of interest, those methods were also used to locate all likely data sources within PubMed that might yield CvT data. Over 24000 publications identified by the method described in Literature Source Data Extraction are available in the **documents** table (see Fig. 2). To increase the accessibility of these sources, they have been tentatively linked to a chemical by searching them for ‘preferred_names’ from DSSTox (EPA’s Distributed Structure-Searchable Toxicity Database)^33^. Almost 20000 of the sources had a linkage to a chemical entity in DSSTox based on this simple search. Although manual curation would be required to confirm the linkages, 1476 of these chemical names match the name or CAS of chemicals listed in TSCA (a list of chemicals produced or imported into the United States, with certain exceptions)^34^ or FIFRA^35^ (a list of chemicals registered as pesticides in the United States), which suggests they may be environmentally relevant; these are marked with a Boolean. Chemical names matching compounds tested under ToxCast^36^ are also marked to support IVIVE research. There are likely to be false positives due to incorrect machine identification of topical chemical names (for example, the word “lead” only sometimes refers to a compound) and false negatives due to the name by which the compound is referred to in the abstract being different from the preferred name, but based on the precision observed in our evaluation of extraction techniques (see the **Technical Validation: Source Identification** section infra), as many as 3116 of these abstracts are true sources of relevant CvT data. Citations for unextracted publications and their tentative chemical mapping is provided as a csv file (CvT_unextracted_sources.csv) on GitHub or in the **documents** table of the database.

### CvT time-series

CvT results consist of the measured *in vivo* concentration-time data points (generally extracted from figures in the paper) resulting from a toxicokinetic study and the experimental details that provide the context for that toxicokinetic curve. Each data point is stored in the **conc_time_values** table as seen in Fig. 2. The **series** table contains details regarding each set of values, and **studies** contains more general information about the pharmacokinetic experiment. Each study is linked to **documents**, which cites the information source.

The original inspiration for the set of contextualizing metadata was based on the test guideline for metabolism and pharmacokinetics released by the U.S. EPA Office of Prevention, Pesticides, and Toxic Substances (OPPTS)^37^. Identifying the set of parameters necessary to properly annotate the extracted CvT data was iterative, with improvements evolving after the review of multiple publications (as the consistency and reporting of study details is highly variable in literature). Below is the set of key study details collected:

- Reference (data source document identification, the name of the figure or table from which the data was gleaned, explanatory notes provided in the source publication)
- Study scenario (test substance, administration route, dose amount, vehicle, and volume, exposure duration, quantity of doses given and their spacing, number of subjects per treatment group, number of treatment groups, fasting status of subjects)
- Subject details (species, type/strain, sex, age, age category, size, and any other description given by source)
- Measurement details (the original time and concentration units, analyte, the medium (tissue, circulatory fluid, etc.) in which the analyte was detected)
- Measurement methods (the limits of detection and/or quantification, the analysis method)
- Curation notes (known assumptions made during the collection process)

During extraction of the data from concentration-time plots, values indicating the extrema of any y-axis (concentration) error bars were captured for some series (collection of error bars started partly through our curation process). These were assumed to indicate the standard deviation. When the standard deviation was calculated in log space, the non-logged values were stored as conc_lower_bound and conc_upper_bound. All data was stored with the intent to maintain the original content with as little alteration as possible in the “*_original” fields and then normalized into corresponding “*_normalized” fields (e.g., see the *conc_units_original* and *conc_units_normalized* fields in the **series** table).

**Fig. 3:**
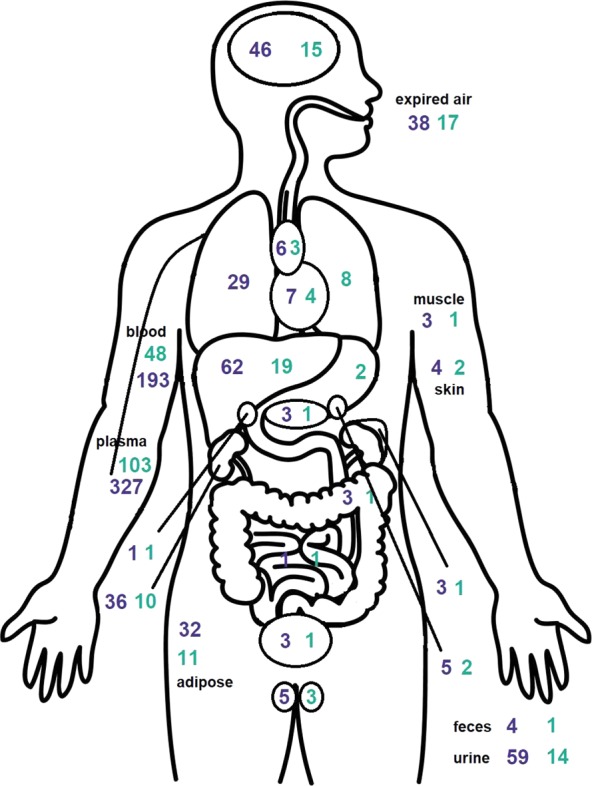
Count of studies (in purple) and test substances (in green) with CvT results in different media (for any species, but represented here on a human body).

The manual extraction of data for the pilot set of chemicals derived from the 2012 TSCA Work Plan in resulted in 291 studies associated with 50 test substances. In total, we have collected CvT data for 144 chemical substances tested in 571 studies resulting in 16267 series. The data collected represent time courses in many different media/tissues, species, and administration routes. The breadth of media/tissue coverage is illustrated in Fig. 3. A table containing the count of studies for each chemical in particular routes and species is contained in the file available on GitHub (routes_and_species_per_chemical.csv).

**Fig. 4:**
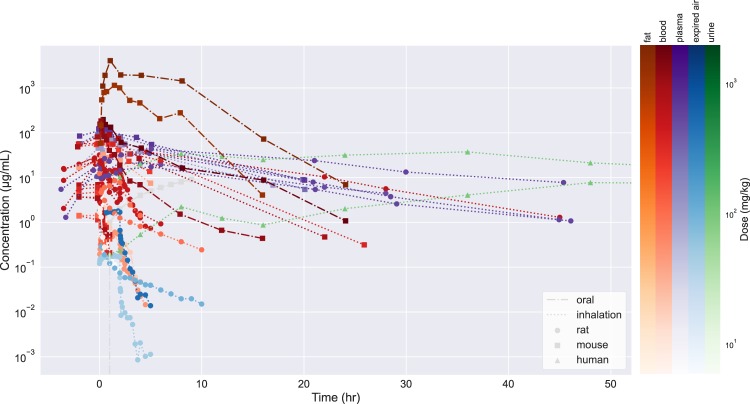
CvT values for trichloroethylene across species, doses, and routes.

To demonstrate the range of CvT data and its variability, Fig. 4 provides CvT values for the most highly represented chemical substance in the database, trichloroethylene. The detailed CvT data is available through the sql dump file provided in the zipfile on figshare or GitHub.

### Toxicokinetic parameters

CvT time-series provided a basis for estimating several TK parameters. These parameters are stored in the **tk_parameters** table, linked to the time-series from which they were derived. A binary flag field “calculated_with_invivopkfit” can be used to separate parameters calculated using the standard CvT analysis package invivopkfit v1.5 (as described in the **Methods** section) and the data collected from the source documents. Parameters include Cmax (the maximum concentration), total clearance, Michaelis-Menten values Km and Vmax, and elimination half-life. Distributions of the generated TK parameters can be seen in Supplementary File 1.

## Technical Validation

### Source identification

Identification of sources proved to be more difficult than expected, leading to a small study to evaluate and validate methods of source selection to improve our workflow efficiency. Our baseline search technique using MeSH terms is documented in **Methods: Literature Sources** above; however, this baseline search proved to have a high false positive rate leading to the review of many articles with no CvT data. In an attempt to reduce the false positive rates observed in our initial review of the identified literature, we devised five data source identification methods for performance comparison on a pseudo-random set of 1000 publications returned by chemical-based querying of PubMed. The set of 1000 test publications was filtered to only the publications with both abstracts and MeSH annotations, which reduced the sample size to 773. The methods applied can be broken down into two classes: MeSH filters and NLC. Two of the MeSH filter approaches were just simple searches for (A) MeSH terms containing “Dose-Response Relationship” (our baseline) or (B) the MeSH term “Pharmacokinetics” within the set of publications.

The next three methods used 611 already-reviewed publications as a training set of documents containing CvT data (positive) and documents with no CvT data (negative) documents. We identified MeSH terms that appeared highly represented (in greater than the square root of that class’s number of articles) in the positive and negative classes. MeSH terms that were highly represented in both positive and negative classes were removed from both. Lists of the identified positive and negative MeSH features are provided in a text supplement on GitHub (tk_enriched_MeSH_terms.csv). For search (C), publications having any positive MeSH feature but zero negative MeSH features were classified as positive. More advanced classification tools were deemed inappropriate for application to MeSH annotations because the number of terms per document was extremely variable. For NLC, models were developed as described in **Methods: Literature Sources** using criteria that either (D) all models or (E) 75% of the models needed to predict the publication as a positive. All papers classified as positive by at least one test method were requested from the EPA library and manually reviewed (283 total). The resulting recall statistics are provided in Table 2. We concluded from these results that Test D was the best data source identification method to apply with our limited curation resources.

**Table 2:** Summary of data source identification test results.

Test	Description	TP	FP	FN	recall	precision	F1 score
A	MeSH: Dose-response	0	48	19	0	0	0
B	MeSH: Pharmacokinetics	9	22	10	0.47	0.29	0.36
C	Positive/negative MeSH	6	220	13	0.32	0.03	0.05
D	100% consensus	14	26	5	0.74	0.35	0.48
E	>75% consensus	19	65	0	1.0	0.23	0.37

### Data validity

In assessing the validity of the data collected, three aspects were considered: limitations in the source material, unreported experimental variability from CvT experiments, and inaccuracy in the collection techniques applied when extracting the data from source content.

First, we considered the limitations in the source material. Source data (being primarily from peer-reviewed literature sources) was generally assumed to be error-free. There were cases where the graphical representation of the data did not unambiguously convey experimental results (e.g., blurry images, concentration points below the graph’s datum). In these cases, the data were extracted to the best of our ability and annotated in the database with a curation note.

The chemical tested or analysed was usually only identified by their name in the source documents. This leaves some room for ambiguity^38^. The source names were mapped to unique substances (designated by DSSTox SIDs) through expert curation.

It is important to note that the time-series data came with the experimental details that Chemicals in unstructured data were usually only identified by their name in the source documents. This leaves some room for ambiguity^38^. Names were mapped to unique substances (designated by DSSTox SIDs) through expert curation.

It is important to note that the experiment details provided with the source data came with varying degrees of completeness. It was common for study details that could be relevant to interpretation and comparison between experiments to be omitted in the source material. For example, the method used to quantify chemicals in media, the recovery amount associated with that method, and its associated limit of quantification were rarely reported in our sources. To give another example, 145 of 263 oral dosing studies did not report whether subjects were fasted. Absence of study details from the database should be considered by the user when accessing the validity of the data for their use cases.

Another notable example of a type of incompleteness is that 5180 of 6769 concentration points representing multiple subjects did not include any estimate of variability. Most of these series had sample sizes of 4, 5, or 6; it is not obvious how relevant a mean of that sample is to population mean behavior. Even when included, it was rarely clear whether error bars represented standard deviation, standard error, or another measure. We consider improved estimates of central tendency through comparison across studies one of the benefits of this data set for PBPK modelers.

Because experimental variability was often underreported in our source material, we analyzed results from a paper that performed the same study several times to serve as controls for different treatment groups on a 25 mg/kg dose of phenytoin^39^, yielding three replicates each of test compound and metabolite results from IV administration and two replicates each from oral administration. We consider this a proxy for the minimum possible amount of variance due to unknown biological differences or other experimental variability, since the replicate series use the same subject pool, same experimenters, same analytical methods, mean concentrations representing a higher-than-average number of test subjects per series (21), and identical quantities of time points over the same study durations. Using the Kolmogorov-Smirnov test (a two-sided test for the null hypothesis that 2 independent samples are drawn from the same continuous distribution) with an alpha of 0.05 as implemented in SciPy^40^, the replicates are affirmed to be from the same distributions for the oral metabolite and IV and oral dosed compound replicates, but not for the IV metabolite studies. AUCs (area under the curve) were calculated in Python 3 using the Numpy function trapz for each extraction set. The standard deviation of the AUCs was converted to a fraction of the total plot space to normalize uncertainties across plots.

$$AUCFracVar=\frac{\sigma AUCs}{{A}_{plot}}$$

**Eqn 1**. Fractional variability between different CvT time-series plots.

The AUCFracVar was around 5% across the case study series. As this is derived from a single set of experiments for a single substance, it is possible that this variability is not representative for other chemicals.

Finally, we sought to evaluate the uncertainty inherent in the use of computer-assisted extraction techniques to collect the CvT time-series data. Specifically, a pseudo-random sample of 13 series (based on the square root of N plus one rule) were re-extracted to characterize differences in measurements due to personal technique, image resolution, calibration, data point size/shape, or other factors: once by the same curator, and once by a newly-trained curator. AUCFracVar between extraction instances was 0.06% with the same curator, and 0.44% with a different curator (with high variation in one series driving almost half of the difference). Since the magnitude of extraction error is less than we expect the error due to experimental or biological variance (based on the case study described above), we conclude that the data was faithfully transcribed from the source material.

## Usage Notes

TK data is often used by researchers to better understand the complex relationship between external exposures and internal doses. Typically, this is done through the development of complex PBPK models that are fit to the data to represent mechanisms of that particular chemical’s absorption, distribution, metabolism, and excretion (ADME). This database provides a trove of structured data that can be used to develop such “bespoke” models thereby acting as an easily accessible resource for such research. However, when attempting to understand the TK of an individual chemical, collection and extraction of the relevant data from literature, while taxing, is not overly burdensome (and review of such literature is often necessary to understand mechanisms that should be considered for that chemical). This database is more tailored to the development and validation of generic TK models. Such models are intended to provide estimates of TK profiles for broad sets of chemicals. Evaluating and improving generic TK models has been inhibited by the lack of structured data sources containing CvT data for large set of chemicals. By crafting the largest set of openly available CvT data, we hope that iterative testing and improvement of generic models will be facilitated.

Another possible use case would be the inclusion of the provided CvT data or derived TK parameters to aid in assessing a chemical’s risk. All data is from peer-reviewed journal articles or the National Toxicology Program, and can be treated with the same general confidence level you would afford any published data. It is important that in such a situation, that the researcher restrict their analysis to datasets from experiments that meet a specific standard; it may be necessary to refer to the source documents to find whether studies meet your criteria. The authors welcome feedback on additional metadata that would be useful for filtering results.

The current dataset contains a large subset of data based on an initial, externally-identified list of chemicals, as well as the easily obtainable already-structured datasets. However, it covers only a small proportion of the extent of the tested chemical space and doesn’t necessarily contain all instances of concentration time-series data for any of the chemicals therein. It can be used to investigate similarities between series, but it is up to each user to assign a significance to the similarities. Likewise, the pharmacokinetic parameters calculated from aggregated series will change based on inclusion of new sets. Researchers using these parameters should develop their interpretation by examining the R code that generated them.

The set of likely CvT sources linked to tested chemicals is intended to be both a resource and a call for help in this effort. Such information can be used to aid users in finding CvT data that has not yet been structured for their own research objectives, but the size of the data extraction task is large and unlikely to be completed by such a small set of researchers. Readers are encouraged to contribute either their own experimental data or experimental data they have extracted from other sources, including as much metadata as possible, in the format shown in a CSV template (CvT_data_template.xlsx) on our GitHub repository.

## Supplementary information

Supplementary File 1

## Data Availability

The Python code to identify possible CvT data sources in literature is available at our GitHub repository. Download of the database is required to train the model.
