# Supplementary figures and images for: Database of pharmacokinetic time-series data and parameters for 144 environmental chemicals
Source: Sci Data. 2020 Apr 20;7:122. doi: 10.1038/s41597-020-0455-1 (PMC7170868; doi:10.1038/s41597-020-0455-1)

Volume of Distribution

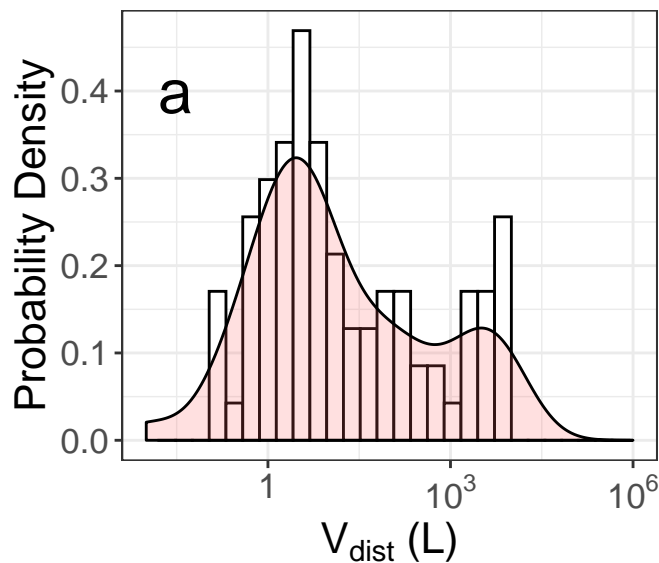

Elimination Rate

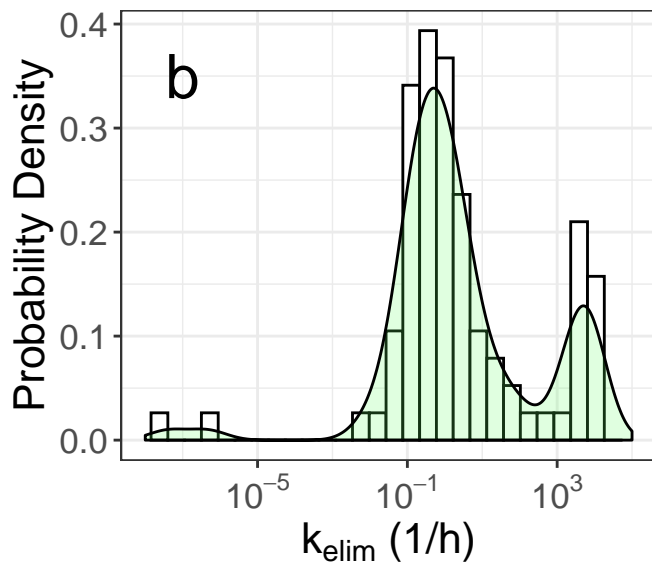

Oral Bioavailability

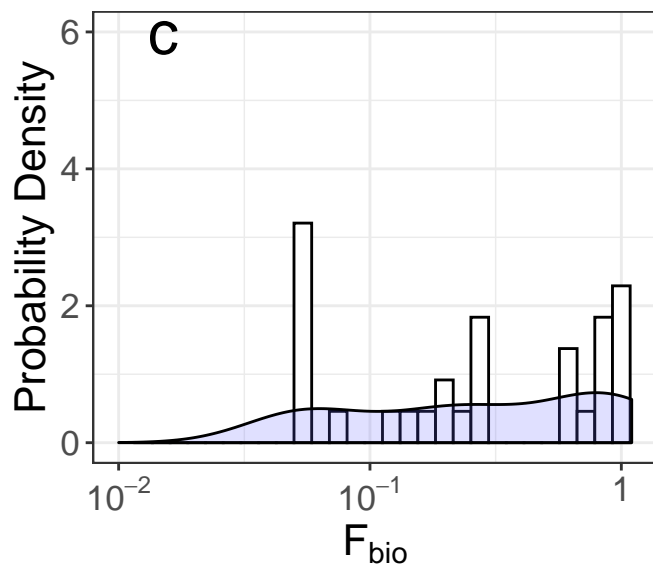

Oral Absorption Rate

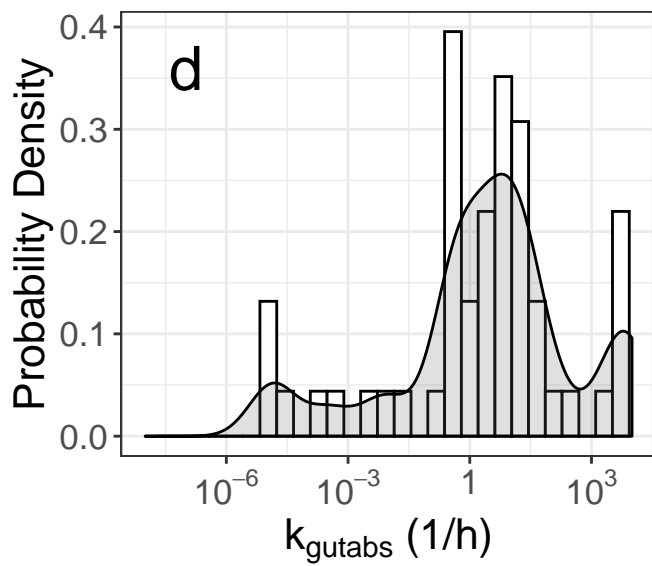

Supplement: Supplementary file 1 [file 41597_2020_455_MOESM1_ESM.pdf]
